# Focusing and alignment of erythrocytes in a viscoelastic medium

**DOI:** 10.1038/srep41162

**Published:** 2017-01-24

**Authors:** Taesik Go, Hyeokjun Byeon, Sang Joon Lee

**Affiliations:** 1 Center for Biofluid and Biomimic Research, Department of Mechanical Engineering, Pohang University of Science and Technology (POSTECH), Pohang, 790-784, South Korea

## Abstract

Viscoelastic fluid flow-induced cross-streamline migration has recently received considerable attention because this process provides simple focusing and alignment over a wide range of flow rates. The lateral migration of particles depends on the channel geometry and physicochemical properties of particles. In this study, digital in-line holographic microscopy (DIHM) is employed to investigate the lateral migration of human erythrocytes induced by viscoelastic fluid flow in a rectangular microchannel. DIHM provides 3D spatial distributions of particles and information on particle orientation in the microchannel. The elastic forces generated in the pressure-driven flows of a viscoelastic fluid push suspended particles away from the walls and enforce erythrocytes to have a fixed orientation. Blood cell deformability influences the lateral focusing and fixed orientation in the microchannel. Different from rigid spheres and hardened erythrocytes, deformable normal erythrocytes disperse from the channel center plane, as the flow rate increases. Furthermore, normal erythrocytes have a higher angle of inclination than hardened erythrocytes in the region near the side-walls of the channel. These results may guide the label-free diagnosis of hematological diseases caused by abnormal erythrocyte deformability.

A detailed understanding of the transport processes (e.g., focusing, separating, sorting, counting, and detection) of particles or cells in flowing fluids is important for various industrial and biomedical applications. The cross-streamline migration of particles induced by hydrodynamic forces of streaming fluids varies depending on the geometry of the channel and on the physicochemical properties of the particles and fluids involved^1^,^2^,^3^,^4^. Many researchers investigated the dynamic behaviors of particles in Newtonian fluid flows^5^,^6^,^7^,^8^,^9^,^10^. In this case, the inertial effect in a laminar flow mainly induces the lateral migration of particles. In flows with Reynolds number (*Re*) ≥ *O*(1), lift forces exerted on particles enable them to move laterally toward equilibrium positions. Dominant lift forces include wall-induced and shear-gradient-induced lift forces. Segre and Silberberg^8^ firstly observed that neutrally buoyant rigid spheres form an annular ring and focus at a radial position of around 0.6 *R (R*: pipe radius). Di Carlo *et al*.^6^ conducted experiments and numerical studies to explain the inertial migration of particles through scaling analyses of shear-gradient-induced and wall-induced lift forces. The inertial migration of cells in Newtonian fluids has been extensively applied for label-free separation by cell size, shape, and deformability^11^,^12^,^13^,^14^,^15^,^16^. However, the pattern of inertial focusing increases in complexity at a high *Re* and attains unfavorable multiple equilibrium positions^17^,^18^.

Fluid viscoelasticity can also induce a transversal force on streaming particles. Recently, particle migration in a viscoelastic fluid has received substantial interest because this phenomenon provides high-quality focusing even at a flow rate one to two orders of magnitude slower than that of inertial focusing. Leshansky *et al*.^19^ observed that particles migrate toward the centerline because of the imbalance in normal stresses exerting on the particle in a slit channel. Elastic focusing of particles in a core stream can be achieved in a specific range of flow rates on the basis of the geometrical parameters and rheological properties of the working fluid. Yang *et al*.^20^ demonstrated particle focusing to the centerline of a square microchannel derived from the combination of the elasticity and inertia of a viscoelastic fluid flow. D’Avino *et al*.^21^ observed particles moving from the wall to the center and proposed a design rule for the viscoelastic focusing of particles in a micropipe flow. As a practical application of elasto-particle migration, particles can be separated depending on their size and shape^20^,^22^,^23^,^24^.

Blood plays an important role in the diagnosis of human diseases. The biochemical and mechanical properties of erythrocytes are highly sensitive to the stages of various hematological diseases^25^. Several hematological diseases, including malaria^26^,^27^, diabetes^28^, sickle cell anemia^29^, and hereditary disorders^30^, modify erythrocyte deformability^31^. Blood cell deformability is an attractive biomarker because conventional biochemical reactions require cumbersome and expensive labeling procedures. Typical measurement techniques for cell deformation include micropipette aspiration^32^, atomic force microscopy (AFM)^33^, optical tweezer use^34^, and quantitative phase imaging (QPI)^27^. However, the result of micropipette aspiration is affected by the size of the pipette. Optical tweezer method requires optical trapping and stretching procedures of an erythrocyte and AFM measurement requires a scanning procedure. QPI needs high magnification and complicated optical set-up for investigating membrane fluctuation of an erythrocyte. Furthermore, these techniques are single cell analyses so they yield low throughputs (1 cell/min ~ 1 cell/s) and requires long time for obtaining statistical results^14^,^25^. The fast and accurate examination of cell deformability is essential for the early diagnosis and enhanced understanding of such hematological diseases.

Digital in-line holographic microscopy (DIHM) has been widely utilized to analyze the dynamic behaviors of various micro-scale objects, including the swimming motions of microorganisms^35^,^36^,^37^,^38^, lateral migrations of spherical particles^9^,^10^,^39^,^40^, and migrations of ellipsoids and red blood cells^41^,^42^. DIHM can provide 3D volumetric information from a single shot of a hologram^43^,^44^. Each image contains about 40 erythrocytes in the field of view of the present study. Considering the frame rate (60 fps), the spatial information about 2500 cells could be acquired for 1 s. After recording a hologram, the 3D positional information of particles located at various in-plane positions and depths can be accurately obtained by varying the reconstruction depth numerically. Therefore, simultaneously detecting numerous particles in a volume of large depth becomes possible without calibration or depthwise scanning procedures. These advantages enable scholars and practitioners to overcome the technological limitations of conventional optical microscopy, stereoscopic microscopy^45^, defocusing^46^ and deconvolution microscopy^47^.

In the present study, we investigated the lateral migration and single-layer focusing of normal and hardened erythrocytes in a rectangular microchannel flow of a viscoelastic fluid. DIHM was employed to obtain 3D positional information and statistics. An elastic aqueous solution of poly(vinyl pyrrolidone) (PVP) with a constant viscosity was used as the suspending medium, and hardened erythrocytes were prepared by formalin treatment. The effects of cell deformability and flow rate on the migration behaviors and orientations of erythrocytes in microchannel flows of elasticity-dominant fluid were compared.

## Results and Discussion

Yang *et al*.^48^ investigated the lateral migrations of deformable and rigid particles in viscoelastic fluid flow of square microchannel. They focused on the difference of focusing position between deformable and rigid particles at low flow rates. Lim *et al*.^49^ recently observed inertia-elastic particle migration in weak viscoelastic flows of square microchannel at extremely high *Re*. They investigated a human leukocyte stretching and the effects of particle size and anisotropy on the lateral focusing. Compared to those previous studies, we three-dimensionally investigated the focusing and orientation of erythrocytes in high viscoelastic flows at relatively low *Re*. The focusing positions of normal and hardened erythrocytes are the same in rectangular microchannel with high aspect ratio. However, vertical concentrations and orientations in the region near the side walls are different according to the deformability.

### Erythrocyte focusing

**Figure 1:**
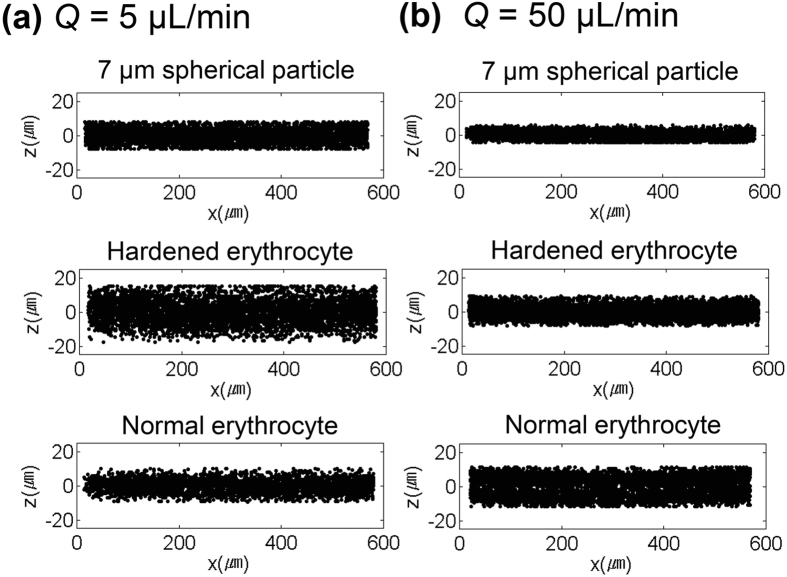
Cross-sectional positions of 7 μm spherical particles, normal erythrocytes, and hardened erythrocytes in the rectangular channel flows of elasticity-dominant fluid (3 wt% PVP solution) **(a)** at *Q* = 5 μL/min (*Re* = 0.02) and **(b)** at *Q* = 50 μL/min (*Re* = 0.2).

We analyzed the spatial focusing of 7 μm spherical particles, particularly hardened and normal erythrocytes in the rectangular microchannel. Figure 1 shows the cross-sectional positions of 20000 micro-scale objects in the rectangular channel flows of the elasticity-dominant fluid (3 wt% PVP solution) at two *Q* values. About 500 holographic images were superimposed in each picture. Although *Q* = 5 μL/min (*Re* = 0.02) was not sufficiently large for inducing inertial focusing, particle-free layers were observed near the top and bottom walls (Fig. 1a). As *Q* is increased to *Q* = 50 μL/min (*Re* = 0.2), additional rigid spheres and hardened erythrocytes migrate from the top and bottom walls (*z* = ±25 μm) to the mid-plane of the channel (*z* = 0 μm). However, the deformable normal erythrocytes fairly disperse at *Q* = 50 μL/min (Fig. 1b).

**Figure 2:**
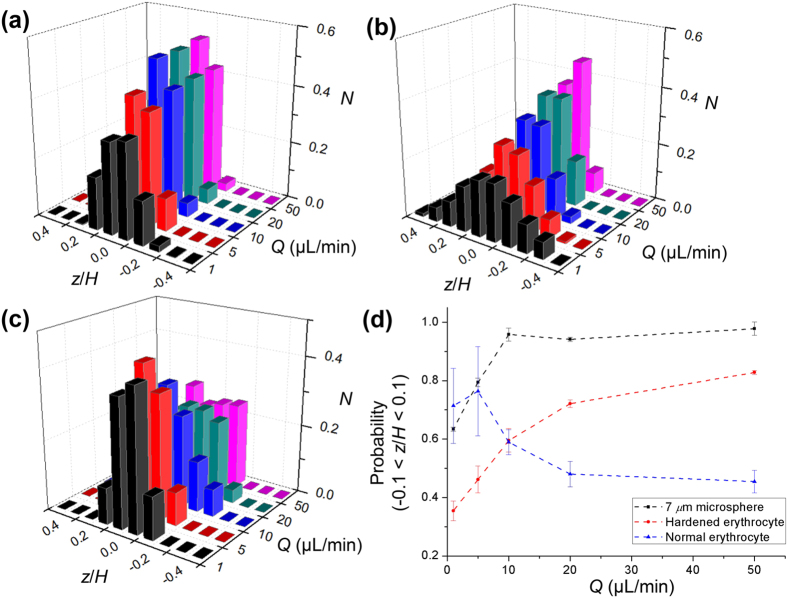
Local concentrations (*N* = *N*_*Δh*_/*N*_*total*_) of **(a)** 7 μm microspheres, **(b)** hardened erythrocytes, and **(c)** normal erythrocytes. **(d)** Variations in local concentration of the three types of microparticles in the channel center (−0.1 < *z*/*H* < 0.1) with flow rate *Q.*

Depth directional local concentrations of microspheres, normal and hardened erythrocytes in the channel cross section were evaluated. The local concentrations (*N* = *N*_*Δh*_*/N*_*total*_) of the three types of microparticles with respect to *Q* are shown in Fig. 2a–c. Herein, *N*_*Δh*_ and *N*_*total*_ represent the number of particles in *Δh* (=0.1 *H, H* = 50 μm) and the total number of particles, respectively. The channel dimension is normalized by the channel height *H*, where *z*/*H* = 0 and ±0.5 indicate the channel center and the top and bottom walls, respectively. The concentration variations of these micro-particles in the channel center (−0.1 < *z*/*H* < 0.1) with *Q* are depicted in Fig. 2d. Single-layer focusing of rigid spheres and hardened erythrocytes is further developed with increasing *Q*. More than 80% of the hardened erythrocytes focus in the channel center plane at *Q* = 50 μL/min. However, the focusing behaviors of normal erythrocytes fairly differ from those of the two other particles tested. At the relatively low flow rate of *Q* ≤ 5 μL/min, more than 70% of the normal erythrocytes focus at the mid-plane of the channel. As *Q* is increased, the local concentration of the normal erythrocytes in the channel center plane gradually decreases.

In a viscoelastic Poiseuille flow, suspended particles principally migrate toward specific positions because of non-uniform normal stress distribution. The nonlinear elastic forces in a viscoelastic fluid are expressed in terms of the first (*N*_*1*_), and the second (*N*_*2*_) normal stress differences, where *N*_*1*_ = σ_*xx*_ − σ_*yy*_ and *N*_*2*_ = σ_*yy*_ − σ_*zz*_ respectively. Here, σ_*ii*_ is the diagonal component of a stress tensor, *x* is the flow direction, *y* is the direction of velocity gradient, and *z* is the direction of vorticity^19^. The contribution of the second (*N*_*2*_) normal stress difference is generally negligible because the magnitude of *N*_*2*_ is much smaller than that of (*N*_*1*_) (|***N***_2_/***N***_1_| < 0.1)^50^. Therefore, the elastic force *F*_*E*_ is mainly generated by the imbalance in the distribution of *N*_*1*_ (*F*_*E*_ ∝ *r*^3^∇*N*_1_, *r*: equivalent particle radius). With the upper convected Maxwell model, *N*_*1*_ can be characterized as 2*μλ*_*r*_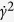. *μ* is the dynamic viscosity and *λ*_*r*_ indicates the relaxation time of a viscoelastic fluid. The upper convected Maxwell model (UCM) was adopted as a constitutive equation for the PVP solution, because it is suitable for predicting viscoelastic fluids with non-zero *N*_*1*_, zero *N*_*2*_, and a constant viscosity^2^. In a low-*Re* region, the lateral migration velocity *V*_*E*_ can be derived by balancing the elastic force and Stokes drag (*F*_*d*_ = 6*πμrV*_*E*_). Consequently, the *F*_*E*_ and the *V*_*E*_ in the Boger fluid (3 wt% PVP solution) can be scaled as follows:


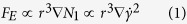



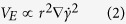


where the square of the shear rate 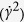 is (*∂U/∂y*)^2^ + (*∂U/∂z*)^2^ for a fully developed flow in the translationally invariant straight channel, and *U* is the flow velocity in the channel. As a result, lateral migration occurs with decreasing absolute value of shear rate.

**Figure 3:**
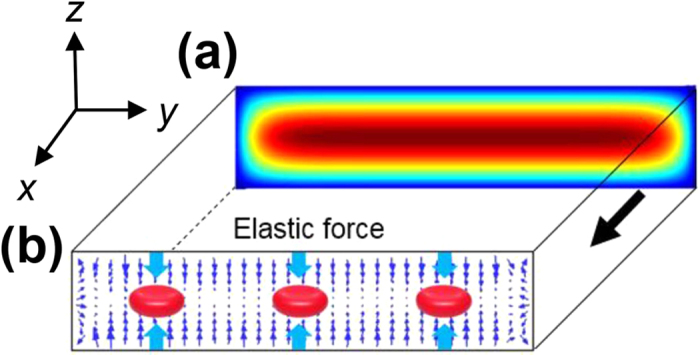
Elastic force exerted on the erythrocytes in a rectangular microchannel of high aspect ratio. **(a)** Streamwise velocity field (*U*). **(b)** Elastic force vectors exerted in the microchannel.

Figure 3 shows the *F*_*E*_ vectors exerted on the particles and erythrocytes in the rectangular microchannel with a high aspect ratio. The fully developed streamwise velocity (*U*) and the square of shear rate are evaluated by 3D finite element simulations using COMSOL Multiphysics software with assuming a constant fluid viscosity. The direction and relative intensity of *F*_*E*_ vectors can be obtained by calculating 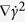 through the information about the square of shear rate. The *F*_*E*_ operates from the walls (high shear rate region) to the channel center plane and four corners (low shear rate region). However, particle focusing at the four corners was not observed in this study because of the size of erythrocytes and the presence of additional wall-induced force^22^. In the rectangular microchannel with a high aspect ratio, the shear rate gradient at the top and bottom walls is much larger than that at the side walls. Therefore, the *F*_*E*_ is mainly governed by the top and bottom walls. As a result, rigid spherical particles and both hardened and normal erythrocytes commonly migrate along the depthwise direction (*z*-axis) and focus in the channel center plane even at low *Re* conditions.

For the rigid spherical particles and hardened erythrocytes, the particle-free layers near the top and bottom walls expand as *Q* is increased (Fig. 2a,b). With increasing *Q*, the high shear rate region near the channel wall is further developed, and the gradient of the shear rate is enhanced. Consequently, the corresponding *F*_*E*_ and *V*_*E*_ increase and additional particles concentrated in the channel center. The local concentration of the hardened erythrocytes in the channel center region is smaller than that of the rigid spherical particles and a high *Q* is necessary for the hardened erythrocytes to achieve the same focused state as that in rigid spherical particles (Fig. 2d). The development of particle focusing and particle concentration in the mid-plane of the rectangular channel is positively correlated with the elastic force *F*_*E*_ and migration speed *V*_*E*_. These results demonstrate that the elastic force *F*_*E*_ on the hardened erythrocytes is weaker than that on the rigid particles. The difference is attributed to the shape and the equivalent radius of the hardened erythrocytes. The equivalent radius of the hardened erythrocytes (*r* = 2.78 μm) is smaller than the radius (3.5 μm) of the particles.

Different from the rigid particles and hardened erythrocytes, the normal erythrocytes receive additional lift force caused by deformation. The additional lift force is induced on an object by the modified flow field due to the asymmetric deformation of the object and the presence of channel wall. It pushes away the object from the channel wall^51^. This lift force contributes to the development of a cell-free layer near the wall even with negligible fluid inertia^51^. The force is generated by the interaction between channel wall and deformable objects, such as deformable drops, vesicles, and erythrocytes. The empirical equation for the lift force *F*_*L*_ for a deformable object located close to the channel wall (*h* ~ *R*) can be expressed as follows:


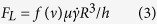


where *f* is determined experimentally. The reduced volume of a particle can be expressed as 
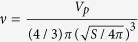
. Here, *V*_*p*_ is the volume, *S* is the surface area, *R* is the equivalent radius of the particle (*R* = (3*V*_p_/4*π*)^1/3^), and *h* is the distance between the particle and the channel wall^51^,^52^. In addition, the lift force *F*_*L*_ can be scaled as ~*R*^4^/*h*^*2*^ if the object is far from the wall (*h* ≫ *R*)^1^. Abkarian *et al*.^52^ reported that a deformable erythrocyte (*ν* = 0.7) receives additional lift force from 31 pN to 155 pN when the distance *h* is 350 nm. However, the wall-induced additional lift force acting on the non-deformed objects located near the channel wall is weak and negligible. In the region with a low *Q*, the deformable erythrocytes can interact with the top and bottom walls because the magnitude of the *F*_*E*_ exerting toward the channel centerline and migration velocity *V*_*E*_ are relatively small. Consequently, more normal erythrocytes concentrated in the channel center region compared with hardened erythrocytes under a low *Q* ( ≤ 5 μL/min).

**Figure 4:**
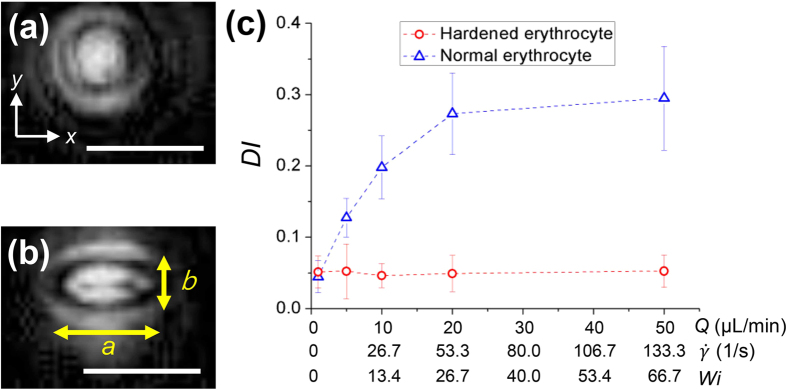
Reconstructed holographic images of normal erythrocytes **(a)** at flow rate *Q* = 1 μL/min and **(b)** *Q* = 50 μL/min. Major axis *a* and minor axis *b* of the ellipsoid were used to define the deformation index (DI). **(c)** Variations in DI of the normal and hardened erythrocytes as a function of *Q*, 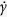, and *Wi.* Scale bars are 10 μm.

In a low-viscosity fluid, erythrocytes flip, tumble, and rotate unsteadily with undergoing minor deformation^53^. However, the shape of an erythrocyte is greatly changed from biconcave to ellipsoidal in a fluid of high viscosity (*μ* ≥ 0.01 Pa∙s)^54^. When a suspension of erythrocytes is subjected to a certain fluid flow, viscous stresses are exerted on the interface, leading to large erythrocyte deformation. Figure 4a,b show the reconstructed holographic images of erythrocytes at two flow rates (*Q* = 1 and 50 μL/min). A normal erythrocyte is deformed significantly and changed into an ellipsoidal shape. We evaluated the average deformation indexes (DI) of both normal and hardened erythrocytes in the rectangular channel as a function of *Q* to quantify the degree of deformation of the erythrocytes in our study (Fig. 4c). The DI was defined as DI = (*a* − *b*)/(*a* + *b*) by using the length of the major axis *a* and minor axis *b* of an ellipsoidal erythrocyte (Fig. 4b). As a result, the deformation of normal erythrocytes non-linearly increases with increasing *Q*, exhibiting a type of strain hardening behavior. However, the hardened erythrocytes nearly maintain a constant shape regardless of *Q*. This phenomenon indicates that the *F*_*E*_ acting on a hardened erythrocyte is only a function of the shear rate gradient, as shown by the nearly fixed radius (equation (1)). However, the shape of a normal erythrocyte changes along the main flow direction (*x*). Although further quantitative analysis about the relationship between the degree of deformation and the elastic force is necessary, the excessive stretching of cells may reduce the hydraulic equivalent radius (*r*) and the elastic force *F*_*e*_ at high flow rate conditions. In addition, Lu *et al*.^24^ reported that elastic force is more dominant on peanut particle of smaller scale. Likewise, the length of the minor axis (*b*) become smaller, as the flow rate increases. Therefore, the deformation of normal erythrocytes adversely affects the focusing in the mid-plane of the rectangular channel under a high flow rate condition of *Q* ≥ 10 μL/min (Fig. 2d).

### Orientation of erythrocytes

Erythrocytes usually exhibit three distinct motions in shear flows depending on fluid viscosity and applied shear rate^53^,^55^. The dynamic behaviors include rotating motion with a periodically varying angular velocity; tank-treading motion, which aligns at a constant angle to the flow direction; and spinning motion, with a symmetry axis aligned with the vorticity axis of the shear field. In low-viscosity fluid media, human erythrocytes have random orientations because of combined motions. Therefore, focusing on such motions in optical imaging is difficult to accomplish. However, in high-viscosity fluid media (*μ* ≥ 0.01 Pa∙s), erythrocytes have certain fixed orientations, which are compensated by a tank-treading motion of the membrane in the cell interior. The shear rate in the microchannel flow is varied from 3/s to 1300/s in this study. Similar ranges of the shear rate and viscosity were handled in the previous studies^54^,^56^.

**Figure 5:**
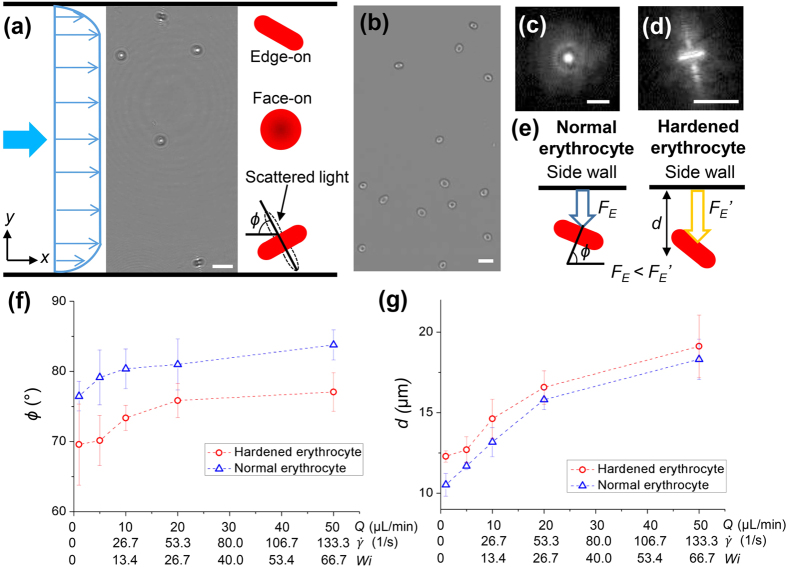
(**a**) Holographic image of normal erythrocytes in the rectangular channel flow at *Q* = 5 μL/min with schematics of the face-on and edge-on orientations of the erythrocytes. **(b)** Optical image of the erythrocytes in the middle plane of the *y*-axis at *Q* = 5 μL/min. Enlarged projection images of the erythrocytes with **(c)** face-on and **(d)** edge-on orientations. **(e)** Schematic of the edge-on orientations of the normal and hardened erythrocytes in the region near the side wall. **(f)** Variations in tilting angle *ϕ* of the hardened and normal erythrocytes as a function of *Q*, 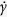, and *Wi.* **(g)** Variations in distance from the edge on erythrocytes to the side wall as a function of *Q*, 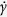, and *Wi.* Scale bars are 10 μm.

Figure 5a represents a typical holographic image of normal erythrocytes in the channel flow at *Q* = 5 μL/min. In this figure, two erythrocyte orientations appear in the rectangular microchannel flow of the viscoelastic liquid. One orientation is the face-on orientation, and the other is the edge-on orientation. The face-on orientation implies that the concave surface of erythrocyte is aligned with the *x*–*y* plane or that the major central axis of the cell is parallel to the *z*-axis. Meanwhile, the edge-on orientation indicates that the major axis of the cell is parallel to the *x*–*y* plane. Figure 5c,d show enlarged projection images of erythrocytes with face-on and edge-on orientations, respectively. The light scattering of the face-on erythrocyte presents a concentric pattern of high intensity in the central region (Fig. 5c). Conversely, the edge-on erythrocyte exhibits a different light scattering pattern. Light scattering from the side surface of the erythrocyte and a straight line along the major axis of the erythrocyte can be noted (Fig. 5d). In consideration of these characteristic light scattering patterns, the erythrocyte orientation was further analyzed. Di Carlo *et al*.^5^ have recently observed the alignment of erythrocytes in a square microchannel when the erythrocytes are fully focused by the inertial effect. The erythrocytes focus at four sites in the cross section for both face-on and edge-on orientations with respect to the top view. However, the origin and appearance of erythrocyte alignment in the rectangular channel fairly deviate from those in the rectangular channel flow of viscoelastic fluids.

Figure 5b shows an optical image of normal erythrocytes in the middle plane of the *y*-axis at *Q* = 5 μL/min. Most of the erythrocytes concentrated in the middle thin layer and they have face-on orientation without tumbling motion. However, the erythrocytes in the viscoelastic fluid flow exhibit an edge-on orientation in the region near the side walls with a certain tilting angle *ϕ* (Fig. 5a). The *ϕ* denotes the angle between the major axis of the cell and the main flow direction. When the distance from the side wall is sufficiently large, the velocity is almost constant in the rectangular microchannel with a high aspect ratio. This finding indicates that the velocity gradient is negligible and that no *F*_*E*_ exists along the *y*-direction. Consequently, the force mainly acts along the *z*-direction. In other words, the erythrocytes in the middle of the *y*-axis only receive the *F*_*E*_ acting from the top and bottom walls of the channel toward the channel center plane. Therefore, an arbitrarily oriented erythrocyte was subjected to the *z*-directional *F*_*E*_. In this case, the erythrocytes reach an equilibrium state when the major axis is perpendicular to the top and bottom walls (Figs 3b and 5b). In the region near the side walls, additional *F*_*E*_ is generated along the *y*-axis because of the existence of the shear rate gradient. Furthermore, the orientation of erythrocytes in Poiseuille flow is closely related with shear stress because erythrocytes undergo varying deformations according to their location in the velocity profile. Therefore, the major axis of the erythrocyte is oriented at a tilting angle *ϕ* due to the existence of velocity difference across the erythrocyte and the short axis is aligned with the vorticity axis (*z*)^56^. As a result, erythrocytes move in the viscoelastic fluid flow with edge-on orientation and a certain tilting angle *ϕ* because of force balance, geometrical constraint, and deformation (Fig. 5e).

Figure 5f shows the variations in the angle of inclination *ϕ* of both hardened and normal erythrocytes as a function of *Q*. The *ϕ* of both types of erythrocytes increases gradually with *Q* and asymptotically approaches to 90°. Korin *et al*.^56^ estimated the tilting angle *ϕ* by modeling erythrocyte motion. The group reported that erythrocytes tend to align with the velocity profile in the channel and align parallel to the wall surface in the near-wall region (*ϕ* → 90°) as the shear rate increases. Our results agree with this previous theoretical study. In addition, the *ϕ* of the deformable normal erythrocytes is larger than that of the hardened erythrocytes. The DI increases with the increase in shear rate proportional to *Q* (Fig. 4c). Therefore, the normal erythrocytes become deformed to a greater extent when they move close to the channel wall. This occurrence implies that the *F*_*E*_ acting on the deformed normal erythrocytes is smaller than that on the hardened erythrocytes in the near side-wall region. The *F*_*E*_ acts on the direction from the side wall toward the middle of the *y*-axis. Hence, the normal erythrocytes move closer to the channel side walls than the hardened erythrocytes at all the *Q* values tested in this study (Fig. 5g). Therefore, the *ϕ* of the hardened erythrocytes is smaller than that of the deformable normal erythrocytes.

In summary, the flow-induced migrations of rigid spherical particles and normal and hardened erythrocytes in the rectangular microchannel flows of a viscoelastic fluid (PVP solution) were experimentally investigated using DIHM. This technique enables the acquisition of accurate information on 3D positions and orientations of such particles in the microchannel. The three types of particles focus in the channel center plane even at low *Q* conditions because of the elasticity effect of the PVP solution. The concave surfaces of most of the normal and hardened erythrocytes in the microchannel with high aspect ratios align with the channel top and bottom walls. This phenomenon is helpful and useful for obtaining optical signatures of erythrocytes with high accuracy and good uniformity. We also noted the effect of the degree of deformability on the lateral focusing and alignment in the rectangular channel flows of viscoelastic fluid. These results can be used to design microfluidic devices for deformability-based cell separation and develop diagnostic tools for hematological diseases.

## Methods

### Sample preparation

The blood was supplied by Korea Red Cross Blood Services. All experimental protocols and procedures were approved by the Ethics Committee of POSTECH. The methods were carried out in accordance with the approved guidelines. The blood samples were initially collected into a preservation solution called citrate phosphate dextrose adenine-1 (CPDA-1) to prevent blood coagulation and biophysical property alteration. The blood was centrifuged, and the buffy coat was aspirated using a micropipette. The separated erythrocytes were rinsed with PBS, centrifuged, separated, and then rinsed again. Finally, the separated erythrocytes were suspended in 3 wt% PVP (M_w_ = 360 kDa, Sigma–Aldrich) diluted in PBS to a hematocrit of 0.5%. The volume fraction of 7 *μ*m is also 0.5%. The low hematocrit was tested in this study to minimize cell–cell interactions. The osmolarity of PBS remained approximately 290 mOsm because of the high molecular weight of PVP. Therefore, the surface-area-to-volume ratio of erythrocytes did not change^54^. To compare the effect of erythrocyte deformability, hardened erythrocytes were prepared separately using a 5% neutral buffered formalin solution. Kuznestosava *et al*.^33^ reported that formalin-treated erythrocytes possess a Young’s modulus 10 times higher than that of normal erythrocytes. The Young’s modulus for normal and hardened erythrocytes are 16.05 ± 2.3 kPa and 119.5 ± 15 kPa, respectively. The hardened erythrocytes were diluted to a density similar to that of normal erythrocytes. All experiments were conducted within 1 h after blood sample extraction from the donor.

### Fluid Rheology Measurements

**Figure 6:**
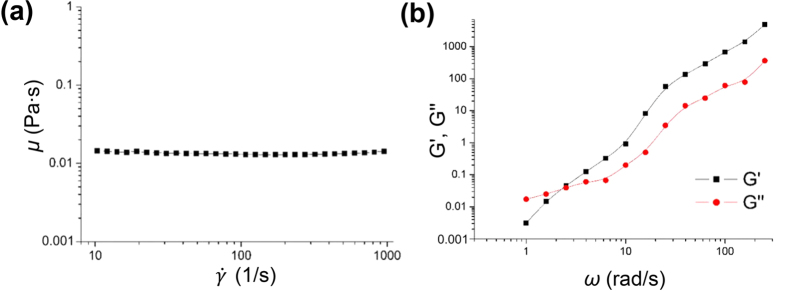
(**a**) Variation in dynamic viscosity (*μ*) of the 3 wt% PVP solution as a function of shear rate. **(b)** Variations in elastic modulus *G*′ and shear modulus *G*″ of the 3 wt% PVP solution used in this study.

First, 3 wt% PVP (M_w_ = 360 kDa; Sigma–Aldrich, USA) was employed as the working fluid. Its rheological properties were measured using a rotational rheometer (HAAKE MARS, Germany) with a cone-plate geometry at 25 °C. Figure 6a shows the variation in dynamic viscosity (*μ*) of the viscoelastic fluid as a function of shear rate. The dynamic viscosity is almost constant (*μ* = 0.015 Pa∙s) in the shear rate range of 10 s^−1^ to 10^3^ s^−1^. This result indicates that the shear thinning effect on the migration of erythrocytes can be ignored. The viscoelastic characteristics of the PVP solution were also measured using a small-amplitude oscillatory test. The frequency responses of the elastic modulus *G*′ and shear modulus *G*″ of the 3 wt% PVP solution are depicted in Fig. 6b. The relaxation time *λ*_*r*_ was roughly estimated as 0.5 s by checking the intersection of both moduli *G*′ and *G*″^22^,^57^. The rheological properties of the PVP solution are similar to those of the Boger fluid, which is an elastic fluid with a constant dynamic viscosity.

### Experimental set-up and conditions

**Figure 7:**
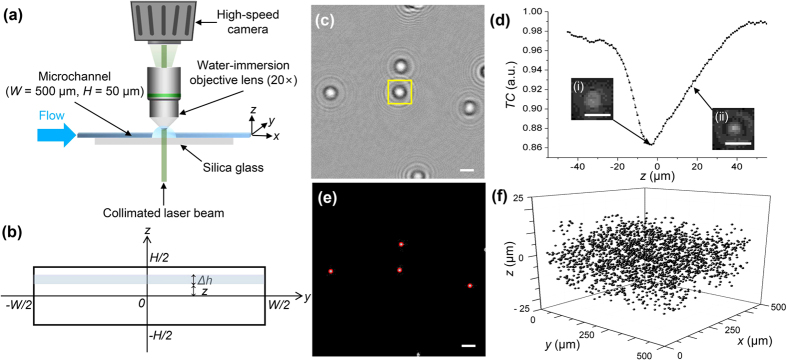
(**a**) Experimental set-up with digital in-line holographic microscopy. **(b)** Coordinate system used for calculating the local concentrations of the microparticles. **(c)** Typical holographic image of erythrocytes with subtracted background image. **(d)** Reconstructed holographic images of an erythrocyte in the yellow square box of **(c)** at (i) *z* = −3 μm and (ii) *z* = 20 μm with a *TC* profile. **(e)** In-plane positions of detected erythrocytes. **(f)** 3D spatial distribution of 3000 erythrocytes in the microchannel. Scale bars are 10 μm.

Single-beam DIHM was conducted to extract the 3D positional information of erythrocytes in the rectangular microchannel^42^. Figure 7a illustrates the experimental set-up, which includes the DIHM optical system. A continuous Nd:Yag laser (*λ* = 532 nm, 100 mW, Crystal Laser, USA) was spatially filtered and collimated. The flowing erythrocytes in the rectangular microchannel were illuminated by the laser beam. A water-immersion objective lens (20×, Nikon, Japan) was used to magnify the generated holograms of erythrocytes. The magnified holograms were then consecutively recorded by a high-speed charge-coupled device (CCD) camera (Ultima APX, Photron, Japan) for 3 min. The spatial resolution was 0.83 μm/pixel in the image plane. A total of 3000 holographic images were then analyzed for each experimental condition to obtain statistically averaged results of the spatial distribution and orientations of the erythrocytes.

Suspensions of normal and hardened erythrocytes were infused into a rectangular microchannel (width *W* = 500 μm, height *H* = 50 μm, aspect ratio *W*/*H* = 10). A transparent rectangular borosilicate glass capillary (VictroCom, USA) of 150 mm in length was used as the microchannel tested in this study. The hydraulic diameter of the micro channel (*D*_*h*_ = *2HW*/(*H* + *W*)) is 90.9 μm. The length (*L*) from the inlet to the measurement section is approximately 9 cm. The ratio of the length *L* to the hydraulic diameter *D*_*h*_ is about 1000, which is sufficient to achieve a fully developed state of particle migration^39^,^40^. Figure 7b shows the coordinate system used to calculate local concentrations. The flow rate *Q* varies in the range 1–50 μL/min. The corresponding Reynolds number (*Re* = *ρU*_*a*_*D*_*h*_/*μ, ρ*: fluid density, *U*_*a*_: average flow velocity, *D*_*h*_: hydraulic diameter, *μ*: dynamic viscosity) is 0.004 ≤ *Re* ≤ 0.2. The Weissenberg number (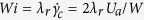, *λ*_r_: relaxation time, 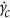: characteristic shear rate) is 1.33 ≤ *Wi* ≤ 66.67. The elasticity number (*El* = *Wi*/*Re*) was also calculated to examine the relative importance of elasticity to inertia. Under the present experimental conditions, *El* was evaluated as *O* (10^2^), indicating the dominance of fluid elasticity.

### Acquisition of 3D distribution of erythrocytes in microchannel

A diffracted object wave is generated when a coherent laser beam irradiates an erythrocyte. The superposition of a diffracted object wave and an unaffected reference wave generates a hologram in the image plane. The time-averaged background image was subtracted from raw holograms to enhance the signal-to-noise ratio of the holographic images. Figure 7c shows a typical background-subtracted holographic image of erythrocytes suspended in the PVP solution. A holographic image was then numerically reconstructed using the angular spectrum method, which is expressed as follows^58^:


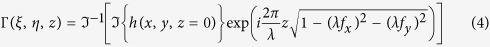


where 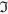 is the fast Fourier transform (FFT) and 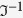 is the inverse FFT. The spatial coordinate (*x, y*) belongs to the holographic image plane, and (*ξ, η*) corresponds to the reconstruction plane. *z* is the distance between the image and reconstructed planes. The function *h*(*x, y*, 0) represents the hologram at image plane *z* = 0. *λ* is the wavelength of the laser beam. *f*_*x*_ and *f*_*y*_ represent spectral coordinates. The magnitude of the convolution 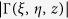 was evaluated to obtain the intensity field of the reconstructed image. The spacing along the depthwise (*z*) direction in the reconstructed image is 1 μm. The reconstructed holographic images of an erythrocyte at two depthwise positions are depicted in Fig. 7d.

The 3D positional information of erythrocytes was consecutively obtained from the reconstructed holographic images of the cells. To find in-plane (*x, y*) positions of erythrocytes, 100 reconstructed cell images were projected to form a single image. Band-pass filtering and thresholding with a maximum pixel intensity of 0.3 were applied to detect the boundaries of the cells and determine their in-plane (*x, y*) positions (Fig. 7e). The area around the in-plane position of each cell was segmented. The depthwise positions (*z*) of erythrocytes were searched by using a refocusing criterion on the basis of the Tamura coefficient (*TC* = [*σ*_*I*_/*μ*_I_]^0.5^), which qualifies image contrast in the segmented area^43^. Herein, *σ*_*I*_ and *μ*_I_ represent the standard deviation and the mean of the intensity distribution in the segmented image, respectively. *TC* has the minimum value at the actual position of the cell (Fig. 7d). Finally, the 3D spatial distribution of 3000 erythrocytes in the rectangular microchannel could be acquired (Fig. 7f).

## Additional Information

**How to cite this article**: Go, T. *et al*. Focusing and alignment of erythrocytes in a viscoelastic medium. *Sci. Rep.* **7**, 41162; doi: 10.1038/srep41162 (2017).

**Publisher's note:** Springer Nature remains neutral with regard to jurisdictional claims in published maps and institutional affiliations.
